# Effects on Strength, Power and Speed Execution Using Exercise Balls, Semi-Sphere Balance Balls and Suspension Training Devices: A Systematic Review

**DOI:** 10.3390/ijerph18031026

**Published:** 2021-01-24

**Authors:** Moisés Marquina, Jorge Lorenzo-Calvo, Jesús Rivilla-García, Abraham García-Aliaga, Ignacio Refoyo Román

**Affiliations:** Facultad de Ciencias de la Actividad Física y del Deporte (INEF—Sports Department), Universidad Politécnica de Madrid, 28040 Madrid, Spain; marquinascience@gmail.com (M.M.); jesus.rivilla@upm.es (J.R.-G.); abraham.garciaa@upm.es (A.G.-A.); ignacio.refoyo@upm.es (I.R.R.)

**Keywords:** instability, resistance training, exercise ball, suspension training, performance

## Abstract

Research in instability has focused on the analysis of muscle activation. The aim of this systematic review was to analyse the effects of unstable devices on speed, strength and muscle power measurements administered in the form of controlled trials to healthy individuals in adulthood. A computerized systematic literature search was performed through electronic databases. According to the criteria for preparing systematic reviews PRISMA, nine studies met the inclusion criteria. The quality of the selected studies was evaluated using STROBE. The average score was 14.3 points, and the highest scores were located in ‘Introduction’ (100%) and ‘Discussion’ (80%). There is great heterogeneity in terms of performance variables. However, instability seems to affect these variables negatively. The strength variable was affected to a greater degree, but with intensities near to the 1RM, no differences are observed. As for power, a greater number of repetitions seems to benefit the production of this variable in instability in the upper limb. Instability, in comparison to a stable condition, decreases the parameters of strength, power, and muscular speed in adults. The differences shown are quite significant in most situations although slight decreases can be seen in certain situations.

## 1. Introduction

Strength training under unstable conditions, as well as destabilizing devices, have gained popularity in the last decade for athletes in order to strengthen core muscles, improve balance, proprioception and increase performance [1,2,3,4]. Muscle power is considered to be one of the main determinants of many short-term explosive sporting events [5]. Power has been defined by different authors throughout history. Bompa [6], defined muscle power as the ability to perform different actions, developing maximum strength in a short period. For this reason, power is a manifestation of the strength that most athletes in different disciplines consider to be of greater importance for the development of certain movements [7,8,9,10,11,12].

A potential reason for similar training-induced adaptations observed after unstable situations compared to stable ones could be related to similar or even higher levels of muscle activation in favour of unstable conditions [13,14]. In the case of the evidence shown concerning the use of unstable situations concerning strength, power and speed, maximum tests have been used to evaluate muscle strength; and tests to determine muscle power (i.e., abdominal power test, medicine ball throwing and different types of jumps) [15,16,17,18,19,20]. However, current results indicate that at least for non-elite athletes, there is a stress/strength and power training intensity pathway that is sufficient to induce positive training adaptations. In their review, Behm and Colado [21] reported that the average strength deficit in unstable situations compared to similar stable exercises was 29%. Furthermore, in healthy young adults, strength training with low loads compared to high loads is equally effective when improving muscle strength [22,23].

Because sport is not usually practised under stable conditions, such as throws, jumps, changes of direction, where the body must be stabilized while a specific action is being performed, training should try to represent the requirements of the specific sport [24,25,26,27]. Also, training under unstable conditions or unbalanced conditions can resemble training and daily activities, providing effective transference [1]. The use of unstable training has been proposed to improve movement specific effect through increased activation of stabilizers and core muscles [14,25,27].

Although initially unstable surface training was reserved for rehabilitation and prevention programs to reduce the rate of injury, due to the proprioceptive overload they provide [28,29,30,31] this type of training is now included in strength and conditioning programs [10,32,33,34]. Currently, the use of these devices has been incorporated into traditional exercises to promote neuromuscular coordination and recruitment but there is controversy regarding the effects of this combination on sports performance and core stability activation [27,35].

There are various ways of generating instability in the performance of exercises but the most common has been through the use of different devices [36,37,38,39]. The use of unstable devices in strength training has led to the development of numerous investigations focused on the analysis of muscle activation [40,41,42,43,44,45,46]. Nowadays, the use of different training methods that retain the stabilising capacity of athletes has become a common and frequent practice. The use of specific devices to create unstable environments, such as exercise or Swiss ball [47], the semi-sphere balance balls, like BOSU [48] and the suspension devices, like TRX [49] have been widely used in sports centres and are widely used throughout the population. Therefore, this review has focused on testing the use of these types of devices and not others, to clarify their influence on sports performance.

### 1.1. Swiss Balls or Exercise Balls

Swiss ball are air-filled balls covered with soft elastic material with a diameter of approximately 35 to 85 cm [47]. The use of unstable surfaces, such as the exercise ball, began to be used in strength and muscle conditioning training as a method of strengthening core, stabilizing muscles, that is the musculature with a deep location which is responsible for a good body posture both in our daily life and in the practice of sports [36,50,51,52]. The response of muscle activity to this unstable surface can be variable and depends on the type of exercise or muscles being tested. Patterns of muscle activation during bench presses have reported variable results based on muscle function. In the stabilising or core muscles, it has been shown that there is an increase in the activation of the internal obliques, the external obliques and the rectus abdominis [25,53,54,55,56] and spinal erectors [56] while having minimal effect on the rectus abdominis [13,56]. In the upper extremities, compared with a stable bench press, a greater increase in anterior deltoid, pectoralis major, triceps and serratus anterior activity has been demonstrated during execution on a stability ball [30,51,55,57,58,59]. However, improvement in muscle activation has not always been evident. In the case of the central or stabilising muscles, other studies have not shown any change in the oblique and internal spinal erectors [60]. In addition, other studies have shown higher data for the stable condition as opposed to the unstable condition, in the main motor musculature responsible for movement, as in the case of the pectorals and triceps in the bench press [43], or no main motor muscle in the shoulder press [59].

### 1.2. Semi-Sphere of Balance

The BOSU Balance Trainer ^®^ (DW Fitness, LLC, Clifton, NJ, USA), or “both sides up” is an exercise device used to improve balance, core muscle or torso strength, and proprioception created for military service veterans [61]. The flat part of the device is a 25-inch platform with two built-in handles, and the other part is an inflatable rubber dome that rises about one foot above the ground. Each side can be used in different ways to create different situations depending on the exercise.

Different studies have analyzed the influence of the semi-sphere ball for training, providing a great variety of results. Authors found increased muscle activity in the rectus abdominis and external oblique in the performance of abdominal plates and gluteal bridge [53]. Anderson and Behm [14] reported increased EMG activity in the vast lateral, soleus and superficial trunk muscles, but not in the femoral biceps when comparing squatting with free weight on a stable versus an unstable surface. In bench press, greater activation of the internal oblique, spinal erector, soleus and biceps femoris was also evident [56]. However, Willardson et al. [35] compared EMG activity in the core performing 50% of 1RM in squats on a stable surface and in a semi-sphere ball and observed no differences between conditions. Authors examined the activity of the brachial triceps, spinal erector, rectus abdominis, internal oblique, and soleus while performing traditional and unstable bending in a single (hands or feet on the unstable surface) or dual (both hands and feet on the unstable surface) instability and found that the dual condition caused the highest percentage of change (>150%) for all muscles analyzed; compared with the other conditions [62].

### 1.3. Suspension Training Devices

A new method available to increase muscle activation is suspension training. This type of training uses the principles of body weight and strength boosting to improve motor unit recruitment [63]. The most applied suspension device is the TRX Home Suspension Training Kit (Fitness Anywhere LLC, San Francisco, CA, USA). In suspension training, a specific device is required to create an unstable condition. This method uses a system of straps with handles on the bottom and which are attached to a single anchor point [64]. Among the different strength training possibilities, suspension training is widely applied in various contexts. It is considered an effective technique for improving neuromuscular activation that precedes the use of heavy loads in traditional exercises [65]. Besides, improvements in speed and strength indicators have been found by the use of suspension training, suggesting increased recruitment of core/stabilizing muscles [66].

Regarding evidence from suspension devices, the effects of usage on both lower body muscle activity [41,67,68] and trunk stabilizing muscle [65,69,70,71] have been investigated. Clear evidence has been established regarding these devices that witnessed increased muscle activation in the stabilizing and synergistic muscles when performing exercises under these conditions [65,69,71,72]. Concerning lower body exercises, very high activation has been shown for the femoral and semitendinous biceps (>90% MVIC) [68], the hamstring, the gluteus maximus, the gluteus medius and the long adductor. Although no significant differences were found in the rectus femoris [41,67]. Also for the Bulgarian squat exercise, no difference in muscle activation was found between the stable and the suspended condition [64]. For the exercises of the upper part of the trunk and the stabilising muscles, they have been studied with the performance of the Push-up exercise. It has been shown that greater muscular activation in the core, rectus abdominis and external oblique muscles [69,73], however, the stable situation reported greater activation for the pectoral and deltoid muscles [65,69,71] the brachial triceps [65] and the clavicular portion of the pectoralis [71]. However, for the frontal plate exercise, Byrne et al. [70] reported no significant differences when studying exercise with suspension devices.

Research has focused on the analysis of muscle activation, determining different considerations, claiming their strengths and weaknesses. However, when the training objective is hypertrophy, or gain in muscle mass, strength, or power, it has not been recommended that the exercises be carried out using unstable situations [5].

As mentioned earlier, muscle activation has been shown to improve the stabilising muscles and reduce the main motor muscles involved in performing the task, but it is not known whether these activations can lead to improved performance.

In terms of the choice of devices, the 3 devices have been widely used for both rehabilitation, proprioception, and development of muscular capacity. With any one of them, there are numerous proposals for working on muscular strength, and all of them focus on the argument of greater activation of the central or core muscles. In fact, the use of suspension devices alone cannot bring about an improvement in strength or power by itself, since one always works with one’s own body weight, without external loads that increase the intensity of the tasks to be carried out. Semi-spherical and exercise ball devices are not only an implement that increases instability, but there are concrete references that indicate that it could be a way to improve strength and power [30,43,59,74]. Although it could have certain limitations, therefore it has been decided to include it, to be able to evaluate all devices and tools that generate instability to a greater or lesser degree.

Therefore, a synthesis of the literature seems necessary to determine whether performing exercises with unstable material provides additional effects on measures of speed, strength and muscle power compared to stable execution. The existing controversy regarding the use and results provided by instability training, and the variability of surfaces, devices, instability positions, samples and exercises generated a heterogeneity of results that makes this review necessary. The aim of this systematic review was to provide a scientifically based study regarding the effects of unstable devices on speed, strength and muscle power measurements administered in the form of controlled trials to healthy individuals in adulthood, apart from muscular activation. It is hypothesized that unstable devices produce similar or not excessively inferior performance improvements to stable conditions because performance with instability is very demanding on the neuromuscular system (i.e., additional stability of joints and posture during exercise is required).

## 2. Materials and Methods

The present research was designed to qualitatively synthesize the available scientific evidence concerning the effect of instability in strength, power, and speed training. The stages of the review procedure and subsequent analysis of the original articles stayed within the guidelines set out in the Preferred Reporting Items for Systematic Reviews and Meta-Analysis (PRISMA) [75] checklist and the Population, Interventions, Comparisons, Outcomes and Study Design (PICOS) question model for the definition of inclusion criteria.

### 2.1. Study Selection and Eligibility Criteria

Primary and original studies to evaluate the strength, power or execution speed in instability were included. Furthermore, studies had to have been published in any language, in peer-reviewed journals with an impact factor included in the Journal Citation Reports of the Web of Science (JCR of WoS) or Scimago Journal & Country Rank (SJR of Scopus) until November 2020.

According to the ‘PICOS’ question model, the inclusion criteria were: (1) ‘Population’: physically active and healthy participants (both men and women) between 18 and 65 years. This age range includes all participants considered to be of adult age; (2) ‘Intervention’: acute training effects on strength, power and/or speed of execution using a Swiss ball, semi-sphere ball or suspension devices; (3) ‘Comparison’: differences in tasks multi-articular upper or lower limb between the execution of exercises in stable conditions and execution in unstable situations; (4) ‘Outcomes’: at least one strength, power and/or speed result had to be reported in the study; (5) ‘Study Design’: descriptive and quasi-experimental research based on a comparison between stable and unstable situations.

The exclusion criteria were: (1) the studies were for intervention periods, randomized control trials, and clinical trials; (2) they included patients or persons with disease or injury; (3) any data about muscle activation, because it is not a performance variable; (4) the subjects were not of adult age (under 18; e.g., children and adolescents and over 65’s as the elderly) (5) the chronic effects of the situations under investigation were assessed; (6) Any other type of unstable device other than Swiss ball, semi-sphere ball or suspension devices, because these are the most frequently used devices; (7) any measurement that includes unilateral exercises or with different types of support such as exercises executed in a monopodal position.

### 2.2. Literature Search

A systematic computerized literature search of the Web of Science, PubMed and EBSCOhost with full text was conducted until November 2020 to capture all relevant articles investigating the effectiveness of instability versus stability. The following Boolean search strategy was applied using the operators ‘AND’, ‘OR’ and ‘NOT’: (‘instability resistance training’ OR ‘instability strength training’ OR ‘free-weight training’ OR ‘suspension training’ OR ‘unstable devices’) AND (‘power’ OR ‘power performance’ OR ‘speed’) AND (‘strength’ OR ‘muscle strength’ OR ‘muscle power’ OR ‘muscular power’) NOT (‘natural surfaces’) AND (‘stability balls’ OR ‘bosu’ OR ‘suspension devices’ OR ‘unstable devices’). The unrestricted language search was limited to the human species and the availability of the full text of original articles reporting on a quasi-experimental trial in an academic journal. Also, we checked the reference lists of each included article and reviewed relevant review articles to identify additional studies suitable for inclusion in the database.

### 2.3. Systematic Review Protocol

**Figure 1:**
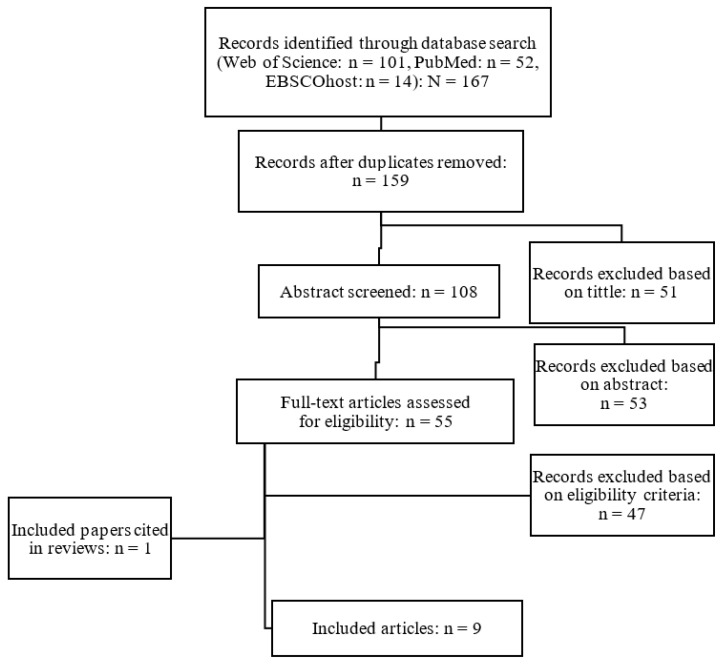
Flow chart illustrating the different phases of search and survey selection.

The authors worked separately and independently to ensure the reliability of the process and the suitable eligibility of the studies. According to the criteria for preparing systematic reviews “Preferred Reporting Items for Systematic Reviews and Meta-Analysis”—PRISMA [75], the protocol carried out in the months of July, August and September 2020 and it was made up of four stages (Figure 1): (1) Identification: the first author (M.M.) found 167 studies in the four digital databases; (2) Screening: the first author (M.M.) eliminated the duplicate files (*n* = 8) and excluded those considered not relevant through a previous reading of the title, abstract and keywords (*n* = 90). Furthermore, the first author (M.M.), jointly with the second (J.L.C.) and third (J.R.G.), rejected the studies linked to the instability according to the exclusion criteria through a full-text reading (*n* = 55); (3) Eligibility: the first (M.M.), second (J.L.C.) and third author (J.R.G.) eliminated full-text studies from the selection process by the eligibility criteria (*n* = 45); (4) Inclusion: the remaining studies (*n* = 8) based on the relationship between the execution of the exercises in a stable condition and their execution in an unstable condition were finally considered. An additional article was identified from the reference lists of included papers and review articles already published [24,63,76,77].

### 2.4. Data Extraction and Management

A standardized form was used to extract data from the studies included in the review for assessment study quality and scientific evidence. Thus, information about (A) ‘authors and year of publication’, (B) ‘sample experience’ (C) ‘sample size and sex’ (number of players, sex), (D) ‘sample characteristics’ (age, height and weight), (E) ‘variable measured’ (strength, power and/or speed), (F) ‘type of exercise’ (G) ‘number of situations’ whether the tasks were executed by comparing only the stable condition vs. the unstable condition or whether more variations were included), (H) ‘device’ (unstable device implemented), (I) ‘training volume’ (number of sets/repeats/rest per exercise), (J) ‘intensity’ (percentage of one maximum repetition (1RM)), (K) ‘strength results’ (maximum strength (e.g., 1RM), mean strength), (L) ‘power results’ (maximum power, mean power and concentric phase power) and (M) ‘speed results’ (maximum and mean speed) were collected. The results data reflect the percentage of decrease or increase in instability concerning stability. If the included studies did not report the results (i.e., means and standard deviations) of the pre-and post-tests, the authors of those studies were contacted.

### 2.5. Study Quality Assessment

The quality of all eligible cross-sectional studies was evaluated using the criteria for strengthening the reporting of observational studies in epidemiology “STROBE” [78]. The following scale was used to rate the quality of studies: (a) good quality (>14 points, low risk of major or minor bias), (b) acceptable quality (7–4 points, moderate risk of major bias), and (c) poor quality (<7 points, high risk of major bias). The score was obtained through the 22 points on the STROBE checklist. Two independent reviewers (M.M. and J.L.) conducted study quality assessment. Rating disagreements were resolved by J.R. and inter-rater reliability calculated.

## 3. Results

### 3.1. Synthesis of Findings (Qualitative Analysis)

Scientific evidence on the sample characteristics (B, C, D), variables (E), exercise and variation (F, G) device used (H) volume and intensity training (I, J) and results for strength, power and speed is shown in Table 1, Table 2 and Table 3. Format and design, including the author and the year of publication, the sample characteristics (overall number, gender, age, height and weight), the variable measured (strength, power and/or speed), type and number of variations, the device used (exercise ball, semi-sphere ball or suspension device), training volume (number of sets/repeats/rest per exercise), intensity training (percentage of one maximum repetition (1RM)), strength results (maximum strength, mean strength), power results (maximum power, mean power and concentric phase power) and speed results (maximum and mean speed) are included.

### 3.2. Sample Characteristics

Table 1 shows scientific evidence on the sample characteristics (B, C, D) and variables (E). Format and design, including the author and the year of publication, the sample characteristics (overall number, gender, age, height, and weight), the variable measured (strength, power and/or speed).

Evaluation of the characteristics of the sample: (B) Experience. The experience of the sample was quite heterogeneous, with the participants standing out trained (*n* = 3; 33.34%); trained in strength without experience in instability (*n* = 2; 22.22%), trained in strength and instability 1 year earlier (*n* = 2; 22.22%); recreational (*n* = 1; 11.11%); no previous experience in strength or instability indicated (*n* = 1; 11.11%); (C) Sex. The distribution of the sample was very unbalanced with more male participants (*n* = 158; 98.14%) than female participants (*n* = 3; 1.86%); (D) Characteristics of the sample. The whole sample was identified as being between 18 (lower limit) and 25 (upper limit) years of age. The height range was identified as 167 cm to 185 cm. The weight range was identified as 79 kg to 88 kg.

### 3.3. Tasks, Devices, and Training Parameters

Table 2 Shows scientific evidence on exercise and variation (F, G) device used (H) volume and intensity training (I, J). Format and design, including type and number of variations, the device used (exercise ball, semi-sphere ball or suspension device), training volume (number of sets/repeats/rest per exercise), intensity training (percentage of one maximum repetition (1RM)).

According to exercise (F): The most evaluated sports task was the “bench press” in five studies (41.67%) and “squat” in four (33.33%). “Deadweight”, “plantar flexions” and “leg extension” were also evaluated (8.33% each of the exercises). (G) The number of situations. The number of comparisons between stable and unstable exercises was 100% of the situations that only compared the stable situation with an unstable one. (H) Type of device. The use of Swiss ball material was 54.55% (*n* = 6); the use of the semi-sphere ball was 36.36% of the studies analysed (*n* = 4). In only one study was a suspension device (TRX) used (*n* = 1; 9.09%). (I) Training volume. The number of series, repetitions and rest was quite heterogeneous. In the case of the series, only two studies are evident, comprising between three and six series. In the case of the repetitions, they varied from isometric execution to 25 repetitions, with the execution of 3–6 repetitions being the most used (60%). In terms of rest, they vary between 3 and 5 min. (J) Training intensity. In the case of the intensity of training, the percentage of load most used was 75% of 1RM (*n* = 3; 30%). 20% of the investigations did not use external load (*n* = 2). The rest of the investigations ranged from maximum repetition to 40% of 1RM.

Table 3 shows scientific evidence on strength results (K), power results (L) and speed results (M). Format and design, including strength results (maximum strength, mean strength), power results (maximum power, mean power and concentric phase power) and speed results (maximum and mean speed).

In performance measures, it can be seen how the use of instability decreases in some cases substantially in relation to the stable condition. Although with loads close to the RM no differences are appreciated. In terms of power, the difference seems to be slighter in stable and unstable condition and even at a higher number of repetitions the instability seems to improve power production. The execution speed also shows a lower production when instability is added.

### 3.4. Strength Results

Concerning the strength parameter with a Swiss ball, the bench press exercise showed 59.4% less isometric strength in instability [13], 5.9% [82], but no differences were found in 1RM [81]. For the lower limb exercises, 70.5% less was evidenced in the unstable condition in leg extension exercises than in the stable condition while the unstable force in plantar flexors was 20.2% less than the stable condition [79]. Also with the same exercise, a decrease with the execution with the semi-sphere ball concerning the stable condition of 19% was evidenced [83]. In the case of the deadweight exercise, the decrease in the maximum isometric contraction between the stable condition and the execution with semi-sphere ball was 10.2% [80]. Regarding suspension training squat exercise with bipodal execution, in the eccentric phase, peak and average force showed a decrease of 46.8% and 13.8% respectively for the lower left limb. In the concentric phase, the use of the suspension training tool caused a decrease of 12.6% in peak force and 12.8% in mean force. For the right lower limb, in the eccentric phase, during execution with the suspension training tool, the force decreased by 42.9% and the mean force by 11.7%. In the concentric phase, during execution with the suspension training tool, the peak and average force decreased respectively by 11.9% and 13.2%. During monopodal execution, the eccentric phase in the left limb, the peak force suffered a decrease of 41.8% and the average force a decrease of 18.1%. In the concentric phase, on the other hand, the use of the suspension training tool caused a decrease of 13.5% in peak force and 15.8% in average force. For the right limb during monopodal execution, in the eccentric phase, the force has decreased by 45.1% and the average force by 17.4%. In the concentric phase, the use of the suspension training tool caused a decrease of 12.4% in the force and 14.3% in the mean force [84].

### 3.5. Power Results

For the variable of power with a Swiss ball, a decrease in the unstable situation of 9.9% concerning the stable situation has been evidenced with the chest press exercise [82], and of 10.3% in the average power, 7.3% in the maximum power and 11.5% in the power exercised in the concentric phase [85]. For the average power, with the execution of 25 repetitions, a decrease of 6.9% was found in the unstable bench press, although in the last three repetitions the average power exercised in the unstable condition was 5.6% higher than the stable condition. On the contrary, among the first three repetitions, the unstable data was 12.9% lower than the stable condition. The power exercised in the concentric phase of the bench press was reported to be 4.6% lower in the unstable bench press, although in the last three repetitions the power exercised in the concentric phase of the unstable condition was 13.2% higher than the stable condition. On the contrary, among the first three repetitions, the unstable data was 13.8% lower than the stable condition [86]. To average power, 25 repetitions showed a decrease of 19.3% in the unstable squat, although in the last three repetitions the average power exercised in the unstable condition was 21.4% higher than the stable condition. On the contrary, among the first three repetitions, the unstable condition was 17.1% lower than the stable condition. The power exercised in the concentric phase of the squat was reported to be 18% lower in the unstable squat, although in the last three repetitions the power exercised in the concentric phase of the unstable condition was 20.6% lower than the stable condition. On the contrary, among the first 3 repetitions, the unstable condition was 16.2% lower than the stable condition [86].

### 3.6. Speed Results

Only one research study has been shown to consider the execution speed of strength exercises measured in instability. In the case of the speed variable with a Swiss ball, there has been a 9.1% decrease in the unstable condition concerning the stable banking press [82].

### 3.7. Study Selection and Assessment (Qualitative Analysis)

The quality analysis (STROBE’ checklist) yielded the following results (Table 4): (a) The quality scores ranged from 13–16; (b) The average score was 14.3 points; (c) Of the 9 included studies, 5 (55.55%) were considered to ‘fair quality’ (13–14 points); and 4 (44.44%) were categorized as ‘good quality’ (15–16 points).

By sections, the highest scores were located in ‘introduction’ (100%) and ‘discussion’ (80%) and among the highest quality studies, items no. 2 (background/rationale); no. 3 (‘Objectives—State specific objectives and/or any pre-specified hypothesis’); no. 6 (participants); no. 7 (variables); no. 8 (‘data source—procedure for determining performance measurement’), no. 11 (‘descriptive results—the number (absolute frequency) or percentage (relative frequency) of participants found in each grouping category and subcategory’); no. 12 (statistical methods); no. 15 (outcome data); no. 16 (main results); no. 18 (‘key results—a summary of key results concerning study objectives’); no. 20 (interpretation) and no. 21 (generalisability) were considered complete (100%), while the most commonly absent or incomplete item (0 points) was found in items no. 9, 10, 13 and 14 (‘main results—a measure of effect size’). The lowest scores were found in the ‘funding’ section (20%).

## 4. Discussion

This is the first systematic review of the literature to examine the effects of instability on measures of muscle strength, power, and speed, administered in the form of quasi-experimental studies in healthy individuals during adulthood.

About the production of force, for the exercises of the upper limb, high decreases in values have been observed for the unstable condition. These differences have ranged from 20 to 75% loss in force development in unstable conditions. According to Kornecki, Kebel, and Siemieński [87], the stabilising function of the skeletal muscles is necessary for the coordinated performance of any voluntary movement, and it significantly influences muscle coordination patterns. Therefore, significant reductions in muscle production probably occurred because the muscles around the shoulder complex needed to give priority to stability over force production. Furthermore, under conditions of instability, the stiffness of the joints that act can limit gains in strength, power and speed of movement [88].

However, in the data evidenced in the study by Koshida et al. [82], the losses in force values are much lower than in the rest of the studies (5.9% loss in instability) compared with 59.4% loss in Anderson and Behm [13] and in the case of Goodman et al. [81], no significant differences are observed. This inconsistency between the previous research can be attributed to the type of muscle contraction, the degree of instability during the recorded task and the equipment. In Koshida et al. [82] the bench press movement was performed in a supine position with the Swiss ball placed in the thoracic area, which provided a broader support base than for other activities performed in a sitting or standing position. Therefore, the instability imposed on the trunk stabilising muscles would probably be less significant than in previous research. Besides, both studies used dynamic contractions with an Olympic bar with weight plates, while Anderson and Behm [13] used isometric contractions with two independent handles held by straps to force the transducers into the ground. The difference in equipment could impose different levels of instability on the shoulder joint and trunk muscles. Although bilateral contractions were performed in both studies, the independence of each hand in the study by Anderson and Behm [13] may have increased the effort required to maintain balance and the need for the muscles to stabilize during maximum isometric contractions, therefore reducing the net force output. In the case of Goodman et al. [81], where no differences were found, it could be due to the use of different loads, since 1RM was used while in Anderson and Behm [13] 75% of 1RM loads were used and in Goodman et al. [81] were used 50% of 1RM. These data could indicate that the percentage of external load can influence the effect of the instability in the training.

In the case of force production in the lower limbs, there have been notable decreases when comparing tasks performed in instability concerning stable conditions. These decreases ranged from 10% to 19%, so it seems that instability affects the upper body more than the lower. With the use of the semi-sphere ball, analysed in terms of strength, with the performance of a dominant hip exercise such as deadweight the decrease in strength was 10.2% [80] while with a dominant knee exercise such as squat it was 19% [83]. This could indicate that certain movements could be affected to a lesser extent depending on the instability. However, as detailed above the methods and loads used were very different. It is noteworthy that many of the studies to check force production in the lower limb using isometric contractions. However, isometric contractions are not usually used in strength training. Despite this, results obtained under isometric contractions have reported that conditions are strongly correlated with dynamic mobility performance [89]. However, due to the isometric test mode, subjects could gradually build up strength while stabilizing and maintaining balance on different surfaces. During the 3 s of maximum effort, the subjects may have been able to stabilize the limbs and trunk and therefore be able to exert a considerable amount of force in unstable conditions. We only know of one study that investigates the production of maximum force in squats on a stable and unstable surface [3]. These researchers used an inflatable balance disk and reported a decrease of approximately 46% in peak force. Although there was a greater decrease in force in that study, it could be attributed to the lack of a familiarization session, which the rest of the studies did consider appropriate.

In terms of strength, there seems to be a differentiation in the data concerning the devices used. When the main movement involves the muscles of the upper body the Swiss ball has been used and in the case of the main movement being performed on the lower body, the semi-sphere ball has been used. The exceptional case was the execution of an exercise such as squatting where the instability with the device in suspension was placed in the upper body. In the case of the Swiss ball, it seems to have a greater influence on the decrease of the force values (differences of 20–75%) compared to the semi-sphere ball (differences between the and 10% and 19%) suspended device (detriments between 12–47%). However, in some cases, the Swiss ball did not produce any differences between the conditions. So, it does not seem to be a determining factor in the case of muscle strength.

Concerning the production of muscular power in the upper limb, decreases in the unstable condition have been observed. Decreases with the unstable condition ranged from 7% to 17%. The data found in the studies that analyzed the bench press with Swiss ball reported a very similar percentage decrease in terms of average power (10.3% [85]; 9.9% [82]; and 12.9% [86]). These small deviations found may again be due to the different percentage of load used (50% vs. 75%) and the different volume of training applied. However, in some situations, instability has produced better power data than a stable condition. These better data have been produced in the last repetitions of the executions with high numbers of repetitions in the exercise (22–25 repetitions). The improvements observed in average power were 5.6% and in the concentric phase 13.2%.

The increase in power observed could be due to previous evidence that has shown that producing a high power output with a light to moderate load would be more effective in developing maximum power than using a heavy load [90,91]. Thus, it appears that such a low rate of reduction may still allow muscle power to be gained from strength exercise in the unstable condition. The mechanism of energy production using the stretch-shortening cycle employs the energy storage capacity of several elastic components and the stimulation of the stretching reflex to facilitate muscle contraction for a minimum period. The concentric muscle action does not occur immediately after the eccentric, the stored energy is dissipated and lost in the form of heat and also the strengthening stretching reflex is not activated. Resistance to instability exercise can compromise the three phases of the stretching-shortening cycle, including the amortization phase. Around this turning point, where the eccentric phase becomes concentric, the maximum force is produced. At the same time, the subjects must stabilize the torso on an unstable surface to provide firm support for the contracted muscles. This additional task can compromise the contraction of the muscles acting on the bar. Their less intense contraction not only prolongs the change of direction of movement but, due to the lower maximum force, impairs the accumulation of elastic energy. The consequence is less speed and power in the subsequent concentric phase [92,93]. However, the subjects of the study by Zemkova et al. [86] were able to produce greater power during the executions on an unstable surface than on a stable one. This higher energy production can be attributed to the so-called ball bounce effect.

In terms of power, there seems to be a differentiation in the data about the devices used. The use of semi-sphere ball seems to have a greater influence on the decrease in power since the detriments with this device varied between 15% and 22%. In the case of the Swiss ball, the decrease in power oscillated between 7% and 14%, with better power data being found in unstable conditions with this device (5–13%). However, when the instability was placed where there was no movement, as in the case of the device in suspension and the squat, improvements of between 5% and 10% in power production were shown. Therefore, placing the instability where the main movement does not occur seems to be a good option for power improvement.

Finally, about the speed of execution, a decrease in the values in unstable conditions in comparison to stable conditions is observed, but the analysis of this variable has hardly been studied. According to Adkin et al. [94], a postural threat in a subject (fear of falling) will lead to a reduction in the magnitude and speed of voluntary movements. Thus, muscle stabilization seems to compromise gains in strength, power and speed of movement [95]. It should also be noted that new patterns of movement are generally learned at low speed, while sport-specific motor actions are executed at high speed [26].

The great heterogeneity in terms of volume and intensity of the load is remarkable. Also, instability seems to affect the force variable to a higher degree, but with intensities close to 1RM no differences are observed. As for power, a greater number of repetitions seems to benefit the production of this variable in instability in the upper limb. Finally, speed has barely been analysed and seems to show losses of speed in instability but not excessively so.

## 5. Implications for Practice

The great heterogeneity found is a limitation of the study, however, the results of this study can be applied in various ways. It would be interesting to include training in instability in athletes trained in this type of situation. All the information about this is with beginner athletes, and what is interesting about the application of instability is the individualization of the subjects. Unstable surfaces can be very interesting tools for optimising training, because although decreases in performance variables have been shown, this may not be the case for experienced athletes. As for integrated work, where besides strength, power and speed, other qualities such as balance can be analysed. Also, the angles with which the work is done in instability are different concerning stable conditions, so that other complementary muscles are worked. Finally, variety in environments, methods and exercises is one of the principles of training and these unstable situations provide it.

The complexity of execution in this type of unstable situation, where the technique can be affected, is noteworthy. For this reason, the level of experience of the athletes is important to be able to apply this type of training. Besides, the population requires the help of a qualified professional who can help and direct the sessions or tasks with this type of device, with an appropriate and individualised programme according to the different users.

## 6. Conclusions

The main findings of this review were that there is great heterogeneity in analysing the acute effects of instability on performance variables. Instability compared to a stable condition decreases the parameters of strength, power, and muscular speed in adults. The differences shown are quite significant in most situations although slight decreases can be seen in certain situations. However, for the upper limb, a greater number of repetitions seems to increase the power values in instability compared to the stable situation. The variables of force, power and speed seem to be affected when instability is implemented. However, it seems necessary to extend the investigation of instability with the performance variables because the results are very heterogeneous and there are no unified criteria to evaluate the different conditions, subjects, tasks and devices.

## Figures and Tables

**Table 1:** Scientific evidence on the sample characteristics (B, C, D) and variables (E).

Reference (A)	Sample			Variables (E)
	Experience (B)	Size and Sex (C)	Characteristics (D)	
Anderson et al. (2004) [13]	Trained in strength, Instability 1 year earlier	10 (M)	a: 26.2 ± 6.0 years	Strength
			h: 177.3 ± 6.0 cm	
			w: 87.3 ± 12.2 kg	
Behm et al. (2002) [79]	Trained	8 (M)	a: 24.3 ± 6.7 years	Strength
			h:178.1 ± 6.1 cm	
			w: 82.3 ± 8.9 kg	
Chulvi-Medrano (2010) [80]	Trained in strength Experience with instability	31 (M)	a: 24.29 ± 0.48 years	Strength
			h: 167.98 ± 8,11 cm	
			w: 79.08 ± 2,37 kg	
Goodman et al. (2008) [81]	Recreational	13 (10 M, 3 W)	a: 24.1 ± 1.6 years	Strength
			h: 176.7 ± 3.0 cm	
			w: 76.0 ± 3.9 kg	
Koshida et al. (2008) [82]	Trained	20 (M)	a: 21.3 ± 1.5 years	Strength
			h: 167.7 ± 7.7 cm	Power
			w: 75.9 ± 17.5 kg	Speed
Saeterbakken & Fimland (2013) [83]	Trained	15 (M)	a: 23.3 ± 2.7	Strength
			h: 181 ± 0.09 cm	
			w: 80.5 ± 8.5 kg	
Sannicandro et al. (2015) [84]	No previous experience in strength or instability is indicated	24 (M)	a: 17.8 ± 0.8 years	Strength Power
			h: 179.1 ± 5.6 cm	
			w: 73 ± 4.9 kg	
Zemkova (2012) [85]	Trained in strength, no experience in instability	16 (M)	a: 23.4 ± 1.9 years	Power
			h: 181.5 ± 6.1cm	
			w: 75.1 ± 6.1 kg	
Zemkova et al. (2017) [86]	Trained in strength, no experience in instability	24 (M)	a: 22.1 ± 1.8 years	Power
			h: 184.5 ± 8.3 cm	
			w: 79.8 ± 9.4 kg	

M = men; W = women; a = age; h = height; w = weight; cm = centimetres; kg = kilograms.

**Table 2:** Scientific evidence on exercise and variation (F, G) device used (H) volume and intensity training (I, J).

Reference (A)	Tasks (F)	Situations (G)	Devices (H)	Volume Training (I)	Intensity Training (J)
Anderson et al. (2004) [13]	Bench Press	Stable and unstable device	Swiss ball	1 set	75% 1 RM
				2 rps	
				2–3 min rest	
Behm et al. (2002) [79]	Leg Extension Plantar Flexors	Stable and unstable device	Swiss ball	1 set	No external load
				2–3 rps isometric	
				3 min rest	
Chulvi-Medrano (2010) [80]	Deadweight	Stable and unstable device	Semi-sphere ball	1 set	70% 1 RM
				6 rps	
				5 min rest	
Goodman et al. (2008) [81]	Bench Press	Stable and unstable device	Swiss ball	1 set	1 RM
				3–6 rps	
				3 min rest	
Koshida et al. (2008) [82]	Bench Press	Stable and unstable device	Swiss ball	1 set	50% 1 RM
				3 rps	
Saeterbakken & Fimland (2013) [83]	Squat	Stable and unstable device	Semi-sphere ball	1 set	20 kg
				Isometrics	
Sannicandro et al. (2015) [84]	Squat	Stable and unstable device	Suspension device	1 set	No external load
				3 rps	
Zemkova (2012) [85]	Bench Press	Stable and unstable device	Swiss ball	6 sets	75% 1 RM
	Squat		Semi-sphere ball	8 rps	
Zemkova et al. (2017) [86]	Bench Press	Stable and unstable device	Swiss ball	1 set	75% 1 RM
	Squat		Semi-sphere ball	25 rps	

rps = repetitions; min = minutes; RM = repetition maximum.

**Table 3:** Scientific evidence on strength results (K), power results (L) and speed results (M).

Reference (A)	Performance Measures		
	Strength Results in Newtons (K)	Power Results in Watios (L)	Speed Results in cm/s (M)
Anderson et al. (2004) [13]	INS (S) = ↓59.4% MIVC		
Behm et al. (2002) [79]	INS (LE-S) = ↓75.4% MVC		
	INS (PF-S) = ↓20.2% MVC		
Chulvi-Medrano (2010) [80]	INS (B) = ↓10.2% MIVC		
Goodman et al. (2008) [81]	INS (S) = No Differences MáxS		
Koshida et al. (2008) [82]	INS (S) = ↓5.9% MS	INS (S) = ↓9.9% MP	INS (S) = ↓9.1% MV
Saeterbakken & Fimland (2013) [83]	INS (B) = −19% MS		
Sannicandro et al. (2015) [84]	INS (EF-LF-T) = ↓13.8 MS		
	INS (EF-LF-T) = ↓46.8 MáxS		
	INS (CF-LF-T) = ↓12.8 MS		
	INS (CF-LF-T) = ↓12.6 MáxS		
	INS (EF-RF-T) = ↓11.7 MS		
	INS (EF-RF-T) = ↓42.9 MáxS		
	INS (CF-RF-T) = ↓13.2 MS		
	INS (CF-RF-T) = ↓11.9 MáxS		
Zemkova (2012) [85]		INS (BP-S) = ↓10.3% MP	
		INS (BP-S) = ↓7.3% Pmáx	
		INS (BP-S) = ↓11.5% CF	
		INS (SQ-B) = ↓15.7% MP	
		INS (SQ-B) = ↓17% Pmáx	
		INS (SQ-B) = ↓15.1% CF	
Zemkova et al. (2017) [86]		INS (BP-S) = ↓12.9% MP (1–3 rps)	
		INS (BP-S) = ↑5.6% MP (22–25 rps)	
		INS (BP-S) = ↓6.9% MP (25 rps)	
		INS (BP-S) = ↓13.8% CF (1–3 rps)	
		INS (BP-S) = ↑13.2% CF (22–25 rps)	
		INS (BP-S) = ↓4.6% CF (25 rps)	
		INS (SQ-B) = ↓17.1% MP (1–3 rps)	
		INS (SQ-B) = ↓21.4% MP (22–25 rps)	
		INS (SQ-B) = ↓19.3% MP (25 rps)	
		INS (SQ-B) = ↓16.2% CF (1–3 rps)	
		INS (SQ-B) = ↓20.6% CF (22–25 rps)	
		INS (SQ-B) = ↓18% CF (25 rps)	

INS = instability; S = Swiss ball; B = Semi-sphere ball; T = Suspension device; LE = leg extension; PF = plantar flexors; EF = eccentric phase; CF = concentric phase; MVIC = maximum voluntary isometric contractions; MVC = maximum voluntary contractions; MS = mean strength; MáxS = maximum strength; LF = left foot; RF = right foot; BP = bench press; SQ = squat; MP = mean power; PMáx = maximum power.

**Table 4:** The study quality analysis (STROBE’ checklist).

Reference	Title and Abstract	Introduction		Methods									Results				Other Analysis	Discussion				Other Information	Strobe Points	Study Quality
	1	2	3	4	5	6	7	8	9	10	11	12	13	14	15	16	17	18	19	20	21	22		
Anderson et al. (2004) [13]	+	+	+	-	-	+	+	+	-	-	+	+	-	-	+	+	-	+	-	+	+	-	13	FAIR
Behm et al. (2002) [79]	+	+	+	-	-	+	+	+	-	-	+	+	-	-	+	+	+	+	-	+	+	-	14	FAIR
Chulvi-Medrano (2010) [80]	+	+	+	+	+	+	+	+	-	-	+	+	-	-	+	+	-	+	+	+	+	-	16	GOOD
Goodman et al. (2008) [81]	+	+	+	+	+	+	+	+	-	-	+	+	-	-	+	+	+	+	-	+	+	-	16	GOOD
Koshida et al. (2008) [82]	+	+	+	+	-	+	+	+	-	-	+	+	-	-	+	+	-	+	+	+	+	-	15	GOOD
Saeterbakken & Fimland (2013) [83]	+	+	+	+	-	+	+	+	-	-	+	+	-	-	+	+	-	+	-	+	+	-	13	FAIR
Sannicandro et al. (2015) [84]	+	+	+	-	-	+	+	+	-	-	+	+	-	-	+	+	+	+	-	+	+	+	15	GOOD
Zemkova (2012) [85]	+	+	+	-	-	+	+	+	-	-	+	+	-	-	+	+	-	+	+	+	+	+	14	FAIR
Zemkova et al. (2017) [86]	+	+	+	-	-	+	+	+	-	-	+	+	-	-	+	+	-	+	-	+	+	-	13	FAIR

## References

[1] Ignjatovic A.M., Radovanovic D.S., Kocić J. (2019). Effects of eight weeks of bench press and squat power training on stable and unstable surfaces on 1RM and peak power in different testing conditions. Isokinet. Exerc. Sci..

[2] Kohler J.M., Flanagan S.P., Whiting W.C. (2010). Muscle activation patterns while lifting stable and unstable loads on stable and unstable surfaces. J. Strength Cond. Res..

[3] McBride J.M., Cormie P., Deane R. (2006). Isometric squat force output and muscle activity in stable and unstable conditions. J. Strength Cond. Res..

[4] Nascimento V.Y.S., Torres R.J.B., Beltrão N.B., dos Santos P.S., Pirauá A.L.T., de Oliveira V.M.A., Pitangui A.C.R., de Araújo R.C. (2017). Shoulder muscle activation levels during exercises with axial and rotational load on stable and unstable surfaces. J. Appl. Biomech..

[5] Wang R., Hoffman J.R., Sadres E., Bartolomei S., Muddle T.W., Fukuda D.H., Stout J.R. (2017). Effects of different relative loads on power performance during the ballistic push-up. J. Strength Cond. Res..

[6] Bompa T.O., Haff G.G. (2009). Periodization: Theory and Methodology of Training.

[7] Arriscado Alsina D., Martínez J. (2017). Muscular strength training in young football players. J. Sport Heal. Res..

[8] Billich R., Stvrtna J., Jelen K. (2014). Optimal velocity to achieve maximum power. KInanthropologica.

[9] Dallas G., Kirialanis P., Mellos V. (2014). The acute effect of whole body vibration training on flexibility and explosive strenght of young gymnasts. Biol. Sport.

[10] Górski M., Starczewski M., Pastuszak A., Mazur-Różycka J., Gajewski J., Buśko K. (2018). Changes of strength and maximum power of lower extremities in adolescent handball players during a two-year training cycle. J. Hum. Kinet..

[11] Pereira L.A., Nimphius S., Kobal R., Kitamura K., Turisco L.A.L., Orsi R.C., Cal Abad C.C., Loturco I. (2018). Relationship between change of direction, speed and power in male and female national olympic team handball athletes. J. Strength Cond. Res..

[12] Spieszny M., Zubik M. (2018). Modification of strength training programs in handball players and its influence on power during the competitive period. J. Hum. Kinet..

[13] Anderson K.G., Behm D.G. (2004). Maintenance of EMG activity and loss of force output with instability. J. Strength Cond. Res..

[14] Anderson K., Behm D.G. (2005). Trunk Muscle Activity Increases With Unstable Squat Movements. Can. J. Appl. Physiol..

[15] Alagesan J. (2012). Effect of Instability Resistance Training of Abdominal Muscles in Healthy Young Females-An Experimental Study. Int. J. Pharm. Sci. Health Care.

[16] Marinković M., Bratić M., Ignjatović A., Radovanović D. (2012). Effects of 8-Week Instability Resistance Training on Maximal Strength in Inexperienced Young Individuals. Serbian J. Sport. Sci..

[17] Sukalinggam C., Sukalinggam G., Kasim F., Yusof A. (2012). Stability ball training on lower back strength has greater effect in untrained female compared to male. J. Hum. Kinet..

[18] Sparkes R., Behm D.G. (2010). Training Adaptations Associated With an 8-Week Instability Resistance Training Program With Recreationally Active Individuals. J. Strength Cond. Res..

[19] Cowley P., Swensen T., Sforzo G. (2007). Efficacy of Instability Resistance Training. Int. J. Sports Med..

[20] Maté-Muñoz J.L., Antón A.J.M., Jiménez P.J., Garnacho-Castaño M.V. (2014). Effects of instability versus traditional resistance training on strength, power and velocity in untrained men. J. Sport. Sci. Med..

[21] Behm D.G., Colado Sanchez J.C. (2013). Instability Resistance Training Across the Exercise Continuum. Sports Health.

[22] Tanimoto M., Sanada K., Yamamoto K., Kawano H., Gando Y., Tabata I., Ishii N., Miyachi M. (2008). Effects of whole-body low-intensity resistance training with slow movement and tonic force generation on muscular size and strength in young men. J. Strength Cond. Res..

[23] Kanehisa H., Nagareda H., Kawakami Y., Akima H., Masani K., Kouzaki M., Fukunaga T. (2002). Effects of equivolume isometric training programs comprising medium or high resistance on muscle size and strength. Eur. J. Appl. Physiol..

[24] Behm D.G., Drinkwater E.J., Willardson J.M., Cowley P.M. (2010). The use of instability to train the core musculature. Appl. Physiol. Nutr. Metab..

[25] Behm D.G., Leonard A.M., Young W.B., Bonsey W.A.C., MacKinnon S.N. (2005). Trunk muscle electromyographic activity with unstable and unilateral exercises. J. Strength Cond. Res..

[26] Behm D.G. (1995). Neuromuscular Implications and Applications of Resistance Training. J. Strength Cond. Res..

[27] Behm D.G., Anderson K.G. (2006). The Role of Instability With Resistance Training. J. Strength Cond. Res..

[28] Chapman D.W., Needham K.J., Allison G.T., Lay B., Edwards D.J. (2007). Effects of experience in a dynamic environment on postural control. Br. J. Sports Med..

[29] Drake J.D.M., Fischer S.L., Brown S.H.M., Callaghan J.P. (2006). Do Exercise Balls Provide a Training Advantage for Trunk Extensor Exercises? A Biomechanical Evaluation. J. Manipulative Physiol. Ther..

[30] Nairn B.C., Sutherland C.A., Drake J.D.M. (2015). Location of Instability During a Bench Press Alters Movement Patterns and Electromyographical Activity. J. Strength Cond. Res..

[31] Paillard T., Margnes E., Portet M., Breucq A. (2011). Postural ability reflects the athletic skill level of surfers. Eur. J. Appl. Physiol..

[32] Hornsby W., Gentles J., MacDonald C., Mizuguchi S., Ramsey M., Stone M. (2017). Maximum Strength, Rate of Force Development, Jump Height, and Peak Power Alterations in Weightlifters across Five Months of Training. Sports.

[33] Prieske O., Muehlbauer T., Borde R., Gube M., Bruhn S., Behm D.G., Granacher U. (2016). Neuromuscular and athletic performance following core strength training in elite youth soccer: Role of instability. Scand. J. Med. Sci. Sport..

[34] Dolezal S.M., Frese D.L., Llewellyn T.L. (2016). The effects of eccentric, velocity-based training on strength and power in collegiate athletes. Int. J. Exerc. Sci..

[35] Willardson J.M., Fontana F.E., Bressel E. (2009). Effect of Surface Stability on Core Muscle Activity for Dynamic Resistance Exercises. Int. J. Sports Physiol. Perform..

[36] Escamilla R.F., Lewis C., Bell D., Bramblet G., Daffron J., Lambert S., Pecson A., Imamura R., Paulos L., Andrews J.R. (2010). Core Muscle Activation During Swiss Ball and Traditional Abdominal Exercises. J. Orthop. Sport. Phys. Ther..

[37] Fredericson M., Moore T. (2005). Muscular Balance, Core Stability, and Injury Prevention for Middle- and Long-Distance Runners. Phys. Med. Rehabil. Clin. N. Am..

[38] Kibler W.B., Press J., Sciascia A. (2006). The Role of Core Stability in Athletic Function. Sport. Med..

[39] Schwanbeck S., Chilibeck P.D., Binsted G. (2009). A Comparison of Free Weight Squat to Smith Machine Squat Using Electromyography. J. Strength Cond. Res..

[40] Saeterbakken A.H., Solstad T.E.J., Behm D.G., Stien N., Shaw M.P., Pedersen H., Andersen V. (2020). Muscle activity in asymmetric bench press among resistance-trained individuals. Eur. J. Appl. Physiol..

[41] Miller W.M., Barnes J.T., Sofo S.S., Wagganer J.D. (2019). Comparison of Myoelectric Activity During a Suspension-Based and Traditional Split Squat. J. Strength Cond. Res..

[42] Saeterbakken A.H., Fimland M.S. (2012). Muscle activity of the core during bilateral, unilateral, seated and standing resistance exercise. Eur. J. Appl. Physiol..

[43] Saeterbakken A.H., Fimland M.S. (2013). Electromyographic activity and 6RM strength in bench press on stable and unstable surfaces. J. Strength Cond. Res..

[44] Saeterbakken A., Andersen V., Brudeseth A., Lund H., Fimland M.S. (2015). The effect of performing bi- and unilateral row exercises on core muscle activation. Int. J. Sports Med..

[45] Dunnick D.D., Brown L.E., Coburn J.W., Lynn S.K., Barillas S.R. (2015). Bench press upper-body muscle activation between stable and unstable loads. J. Strength Cond. Res..

[46] Gonzalo-Skok O., Tous-Fajardo J., Suarez-Arrones L., Arjol-Serrano J.L., Casajús J.A., Mendez-Villanueva A. (2016). Single-leg power output and between-limbs imbalances in team-sport players: Unilateral versus bilateral combined resistance training. Int. J. Sports Physiol. Perform..

[47] Jakubek M.D. (2007). Stability Balls: Reviewing the Literature Regarding Their Use and Effectiveness. Strength Cond. J..

[48] Ruiz R., Richardson M.T. (2005). Using a Domed Device. Strength Cond. J..

[49] Bettendorf B. (2010). TRX Suspension Training Bodyweight Exercises: Scientific Foundations and Practical Applications.

[50] Imai K., Keele L., Tingley D. (2010). A general approach to causal mediation analysis. Psychol. Methods.

[51] Lehman G.J., Gilas D., Patel U. (2008). An unstable support surface does not increase scapulothoracic stabilizing muscle activity during push up and push up plus exercises. Man. Ther..

[52] Vera-Garcia F.J., Grenier S.G., McGill S.M. (2000). Abdominal muscle response during curl-ups on both stable and labile surfaces. Phys. Ther..

[53] Czaprowski D., Afeltowicz A., Gebicka A., Pawłowska P., Kedra A., Barrios C., Hadała M. (2014). Abdominal muscle EMG-activity during bridge exercises on stable and unstable surfaces. Phys. Ther. Sport.

[54] Feldwieser F.M., Sheeran L., Meana-Esteban A., Sparkes V. (2012). Electromyographic analysis of trunk-muscle activity during stable, unstable and unilateral bridging exercises in healthy individuals. Eur. Spine J..

[55] Marshall P.W.M., Murphy B.A. (2006). Increased Deltoid and Abdominal Muscle Activity During Swiss Ball Bench Press. J. Strength Cond. Res..

[56] Norwood J.T., Anderson G.S., Gaetz M.B., Twist P.W. (2007). Electromyographic activity of the trunk stabilizers during stable and unstable bench press. J. Strength Cond. Res..

[57] Barros Beltrão N., Torres Pirauá A.L. (2017). Analysis of muscle activity during the bench press exercise performed with the pre-activation method on stable and unstable surfaces. Kinesiology.

[58] de Oliveira A.S., de Morais Carvalho M., de Brum D.P.C. (2008). Activation of the shoulder and arm muscles during axial load exercises on a stable base of support and on a medicine ball. J. Electromyogr. Kinesiol..

[59] Uribe B.P., Coburn J.W., Brown L.E., Judelson D.A., Khamoui A.V., Nguyen D. (2010). Muscle Activation When Performing the Chest Press and Shoulder Press on a Stable Bench vs. a Swiss Ball. J. Strength Cond. Res..

[60] Lehman G.J., Hoda W., Oliver S. (2005). Trunk muscle activity during bridging exercises on and off a swissball. Chiropr. Osteopat..

[61] Brooks D., Brooks C. (2002). BOSU Balance Trainer: Integrated Balance Training.

[62] Anderson G.S., Gaetz M., Holzmann M., Twist P. (2013). Comparison of EMG activity during stable and unstable push-up protocols. Eur. J. Sport Sci..

[63] Aguilera-Castells J., Buscà B., Fort-Vanmeerhaeghe A., Montalvo A.M., Peña J. (2020). Muscle activation in suspension training: A systematic review. Sport. Biomech..

[64] Aguilera-Castells J., Buscà B., Morales J., Solana-Tramunt M., Fort-Vanmeerhaeghe A., Rey-Abella F., Bantulà J., Peña J. (2019). Muscle activity of Bulgarian squat. Effects of additional vibration, suspension and unstable surface. PLoS ONE.

[65] Snarr R.L., Esco M.R. (2013). Electromyographic comparison of traditional and suspension push-ups. J. Hum. Kinet..

[66] Coswig V.S., Dall’Agnol C., Del Vecchio F.B. (2016). Anthropometric measurements usage to control the exercise intensity during the performance of suspension rowing and back squats. Rev. Andaluza Med. Deport..

[67] Krause D.A., Elliott J.J., Fraboni D.F., McWilliams T.J., Rebhan R.L., Hollman J.H. (2018). Electromyography of the hip and thigh muscles during two variations of the lunge exercise: A cross-sectional study. Int. J. Sports Phys. Ther..

[68] Malliaropoulos N., Panagiotis T., Jurdan M., Vasilis K., Debasish P., Peter M., Tsapralis K. (2015). Muscle and intensity based hamstring exercise classification in elite female track and field athletes: Implications for exercise selection during rehabilitation. Open Access J. Sport. Med..

[69] Borreani S., Calatayud J., Colado J.C., Moya-Nájera D., Triplett N.T., Martin F. (2015). Muscle activation during push-ups performed under stable and unstable conditions. J. Exerc. Sci. Fit..

[70] Byrne J.M., Bishop N.S., Caines A.M., Crane K.A., Feaver A.M., Pearcey G.E.P. (2014). Effect of Using a Suspension Training System on Muscle Activation During the Performance of a Front Plank Exercise. J. Strength Cond. Res..

[71] Calatayud J., Borreani S., Colado J.C., Martin F., Batalha N., Silva A. (2014). Muscle activation differences between stable push-ups and push-ups with a unilateral v-shaped suspension system at different heights. Motricidade.

[72] Snarr R.L., Hallmark A.V., Nickerson B.S., Esco M.R. (2016). Electromyographical comparison of pike variations performed with and without instability devices. J. Strength Cond. Res..

[73] Cugliari G., Boccia G. (2017). Core muscle activation in suspension training exercises. J. Hum. Kinet..

[74] Nairn B.C., Sutherland C.A., Drake J.D.M. (2017). Motion and muscle activity are affected by instability location during a squat exercise. J. Strength Cond. Res..

[75] Moher D., Shamseer L., Clarke M., Ghersi D., Liberati A., Petticrew M., Shekelle P., Stewart L.A. (2015). Preferred reporting items for systematic review and meta-analysis protocols (PRISMA-P) 2015 statement. Syst. Rev..

[76] Behm D.G., Muehlbauer T., Kibele A., Granacher U. (2015). Effects of strength training using unstable surfaces on strength, power and balance performance across the lifespan: A systematic review and meta-analysis. Sport. Med..

[77] Tan B. (1999). Variables to Optimize Maximum Strength in Men: A Review. J. Strength Cond. Res..

[78] Vandenbroucke J.P., Von Elm E., Altman D.G., Gøtzsche P.C., Mulrow C.D., Pocock S.J., Poole C., Schlesselman J.J., Egger M. (2007). Strengthening the Reporting of Observational Studies in Epidemiology (STROBE): Explanation and elaboration. PLoS Med..

[79] Behm D., Anderson K.G., Curnew R.S. (2002). Muscle force and activation under stable and unstable conditions. J. Strength Cond. Res..

[80] Chulvi-Medrano I., García-Massó X., Colado J.C., Pablos C., de Moraes J.A., Fuster M.A. (2010). Deadlift muscle force and activation under stable and unstable conditions. J. Strength Cond. Res..

[81] Goodman C.A., Pearce A.J., Nicholes C.J., Gatt B.M., Fairweather I.H. (2008). No difference in 1RM strength and muscle activation during the barbell chest press on a stable and unstable surface. J. Strength Cond. Res..

[82] Koshida S., Urabe Y., Miyashita K., Iwai K., Kagimori A. (2008). Muscular outputs during dynamic bench press under stable versus unstable conditions. J. Strength Cond. Res..

[83] Saeterbakken A.H., Fimland M.S. (2013). Muscle force output and electromyographic activity in squats with various unstable surfaces. J. Strength Cond. Res..

[84] Sannicandro I., Cofano G., Rosa A.R. (2015). Strength and power analysis in half squat exercise with suspension training tools. J. Phys. Educ. Sport.

[85] Zemková E., Jeleň M., Kováčiková Z., Ollé G., Vilman T., Hamar D. (2012). Power outputs in the concentric phase of resistance exercises performed in the interval mode on stable and unstable surfaces. J. Strength Cond. Res..

[86] Zemkova E., Jelen M., Radman I., Svilar L., Hamar D. (2017). L’effetto delle condizioni di sollevamento stabili e instabili sulla forza muscolare e sul tasso di affaticamento durante esercizi di resistenza. Med. Dello Sport.

[87] Kornecki S., Kebel A., Siemieński A. (2001). Muscular co-operation during joint stabilisation, as reflected by EMG. Eur. J. Appl. Physiol..

[88] Carpenter M., Frank J., Silcher C., Peysar G. (2001). The influence of postural threat on the control of upright stance. Exp. Brain Res..

[89] Stone M.H., Sanborn K., O’Bryant H.S., Hartman M., Stone M.E., Proulx C., Ward B., Hruby J. (2003). Maximum Strength-Power-Performance Relationships in Collegiate Throwers. J. Strength Cond. Res..

[90] Häkkinen K. (1989). Neuromuscular and hormonal adaptations during strength and power training. A review. J. Sports Med. Phys. Fitness.

[91] Wilson G.J., Newton R.U., Murphy A.J., Humphries B.J. (1993). The optimal training load for the development of dynamic athletic performance. Med. Sci. Sports Exerc..

[92] Komi P.V., Bosco C. (1978). Utilization of stored elastic energy in leg extensor muscles by men and women. Med. Sci. Sports.

[93] González Badillo J.J., Gorostiaga Ayestarán E. (1995). Fundamentos del Entrenamiento de La Fuerza. Aplicación al Alto Rendimiento Deportivo.

[94] Adkin A.L., Frank J.S., Carpenter M.G., Peysar G.W. (2002). Fear of falling modifies anticipatory postural control. Exp. Brain Res..

[95] Kornecki S., Zschorlich V. (1994). The nature of the stabilizing functions of skeletal muscles. J. Biomech..

